# 3D-Printed Hybrid Collagen/GelMA Hydrogels for Tissue Engineering Applications

**DOI:** 10.3390/biology11111561

**Published:** 2022-10-25

**Authors:** Anushree Nagaraj, Alaitz Etxabide Etxeberria, Rafea Naffa, Ghada Zidan, Ali Seyfoddin

**Affiliations:** 1 Drug Delivery Research Group, School of Science, Auckland University of Technology, Auckland 1010, New Zealand; 2 BIOMAT Research Group, University of the Basque Country (UPV/EHU), Escuela de Ingeniería de Gipuzkoa, Plaza de Europa 1, 20018 Donostia-San Sebastián, Spain; 3 New Zealand Leather & Shoe Research Association, Palmerston North 4472, New Zealand

**Keywords:** bioprinting, bovine collagen, gelatin methacrylate, hybrid hydrogels, ovine collagen, tissue engineering, water-soluble collagen

## Abstract

**Simple Summary:**

Incorporating natural polymers along with semi-synthetic gelatin methacrylate (GelMA) is known to improve the mechanical properties of developed hybrid hydrogels. Here, we provide a novel comparison of bioprinted GelMA hybrid hydrogel meshes with different concentrations of collagen extracted from bovine hide and ovine skin by assessing their physicochemical characterizations. No previous work has reported the incorporation of ovine collagen with GelMA to develop hybrid hydrogels. Furthermore, different parameters of extrusion-based bioprinting of the inks, and their printing fidelity were investigated. The maximum percentage of collagen that could be incorporated into the meshes was identified as 1%, as these meshes showed good shape fidelity with stable degradation rates. The results indicate these hydrogel meshes may be suitable for specific tissue engineering applications depending on the source of collagen used. Hybrid meshes with ovine (water-soluble) were found to possess properties that could make them suitable for bone tissue engineering applications. Similarly, results indicated that hybrid meshes with bovine collagen could be used for corneal, cartilage, and various soft tissue engineering applications.

**Abstract:**

Bioprinting is an emerging technology involved in the fabrication of three-dimensional tissue constructs for the repair and regeneration of various tissues and organs. Collagen, a natural protein found abundantly in the extracellular matrix of several tissues, can be extracted from collagen-rich tissues of animals such as sheep, cows, rats, pigs, horses, birds, and marine animals. However, due to the poor printability of collagen bioinks, biocompatible collagen scaffolds that mimic the extracellular matrix (ECM) are difficult to fabricate using bioprinting techniques. Gelatin methacrylate (GelMA), a semi-synthetic polymer with tunable physical and chemical properties, has been found to be a promising biomaterial in various bioprinting applications. The printability of collagen can be improved by combining it with semi-synthetic polymers such as GelMA to develop hybrid hydrogels. Such hybrid hydrogels printed have also been identified to have enhanced mechanical properties. Hybrid GelMA meshes have not previously been prepared with collagen from ovine sources. This study provides a novel comparison between the properties of hybrid meshes with ovine skin and bovine hide collagen. GelMA (8% *w/v*) was integrated with three different concentrations (0.5%, 1%, and 2%) of bovine and ovine collagen forming hybrid hydrogels inks that were printed into meshes with enhanced properties. The maximum percentage of collagen suitable for integration with GelMA, forming hybrid hydrogels with a stable degradation rate was 1%. The water-soluble nature of ovine collagen promoted faster degradation of the hybrid meshes, although the structural crosslinking was identified to be higher than bovine hybrid meshes. The 1% bovine collagen hybrid meshes stood out in terms of their stable degradation rates.

## 1. Introduction

Severe damage to the tissues/organs of our body requires the use of tissue engineering to restore/replace damaged tissues by developing functional tissue constructs. Biomaterials, which mimic the natural tissue components, are used for fabricating scaffolds that support the growth and differentiation of cells to replace/restore damaged tissues [1]. Hydrogels, which are made up of a network of crosslinked polymers that allow them to swell in water, are one of these scaffolds. The properties of the polymer used, as well as the nature and degree of crosslinking of these polymers, significantly influence the swelling characteristics of the hydrogels [2,3]. Hydrogels lay out a soft tissue environment and provide ease for the exchange of nutrients. The advantages of hydrogels include excellent biocompatibility, tunable scaffold structure, and an easy-to-control structural framework due to its high-water content that resembles the natural ECM [4,5]. Hydrogels provide excellent scaffolds in cartilage, bone, nerve, and corneal tissue engineering [6,7,8,9].

Bioprinting is a 3D printing technique specific to printing biological tissues. It involves the sequential deposition of polymeric materials, either alone or with cells incorporated, in a layer-by-layer fashion to construct predefined three-dimensional structures. The cells can also be seeded on the scaffold after it has been printed [10,11]. The most commonly used bioprinting techniques include inkjet, extrusion-based printing, and laser-assisted printing. Bioprinting techniques can print tissues with high speed and accuracy [12].

Extrusion-based printing is an extremely attractive 3D bioprinting technique, which utilizes pressure to extrude the biomaterials from a nozzle. The advantages of this technique include its ability to use various materials extruded via different nozzles at the same time and to maintain a homogenous distribution of the printed material [13,14,15]. This bioprinting technique is found to primarily depend on the structural and rheological properties of the polymers used at the printing temperature [16]. The structure of the polymer and its viscosity were found to be crucial properties dictating the printability of the polymers used [17,18]. Polymers with long side chains and high viscosity were found to require a higher printing pressure in order to be extruded from the printing nozzles [19,20].

To date, many biomaterials, including natural, synthetic, and even hybrid materials, have been researched for use as scaffolds for various tissues. Although synthetic biomaterials such as polyacrylamide, polyethylene glycol, poly 2-hydroxyethyl methacrylate, polyvinyl alcohol, polymethylmethacrylate, polyurethane, and polycaprolactone were shown to have good mechanical strength, they failed to support cellular adhesions [21,22,23]. Such materials also lack intrinsic signals, which are essential for recognition by cells and inducing cellular differentiation [24]. Furthermore, the degradation of synthetic materials such as polyglycolic acid was found to release acidic products having the potential to damage the cells [25]. Hence, natural materials including protein-based materials (e.g., collagen), polysaccharide-based materials (e.g., cellulose), and decellularized materials (e.g., amniotic membranes), which resemble native human tissues are being researched [21].

Collagen, a protein secreted by fibroblasts and epithelial cells, is abundantly present in the extracellular matrix of many soft and hard tissues of the human body. It accounts for 25–30% of the human body’s dry weight and has been found to provide and maintain support to a wide variety of tissues. Collagen is known to be a promising biomaterial for tissue engineering applications, as it possesses low immunogenicity, has excellent biocompatibility and biodegradability, and is found to promote cellular adhesion, migration, and growth [26,27,28]. Tissue engineering applications were found to use collagen from a wide range of sources including bovine, ovine, porcine, equine, avian marine, and recombinant sources [27,29,30,31,32,33]. Collagen from bovine and ovine sources can be isolated from collagen-rich tissues, such as the Achilles tendon, pericardium, bone, small intestine, and skin [34,35].

Thermal stability, crosslinking density, viscosity, and other properties of collagen scaffolds were found to depend on the age, type, and location of the tissue. Collagen extracted from younger animal tissues was found to be water-soluble. The solubility of the collagen in water was found to decrease with the increasing age of the animals [36]. Such soluble polymers have been found to have lower sol fraction (%) and higher degradation rates [37,38]. A study by Ghodbane and Dunn (2016) showed that ovine (sheep) tendon collagen scaffolds had better mechanical strength, excellent compressive properties, and were highly resistant to enzymatic digestion when compared to porcine and bovine tendon collagen scaffolds, both of which had comparable properties. The authors successfully demonstrated that there are differences in the properties of collagen depending on their sources [39]. Scaffolds fabricated using bovine hide collagen were found to have better mechanical properties than porcine skin collagen scaffolds, which was attributed to the different structural fiber organizations of the collagen [40]. These mechanical properties, however, are not sufficient for the use of the developed collagen-only scaffolds in tissue engineering applications. Furthermore, scaffolds comprising bovine collagen from the Achilles tendon were found to have better stress-strain characteristics (i.e., it was more durable), higher thermostability and crosslinking ability, and was stronger than equine tendon collagen scaffolds [41]. To our knowledge, no previous study has compared the properties of collagen scaffolds fabricated from bovine hide and ovine skin. 

Depending on the source and type of tissue, the application of collagen in different parts of the body varies. Collagen extracted from the skin of fetal bovine, *Coryphaena hippurus* (marine source), and ovine tendons were found suitable for regenerating tendon and skin, cartilage tissues, and deep skin wounds, respectively [42,43,44]. Collagen scaffolds developed by bioprinting from various sources have been employed in several tissue engineering applications, including nerve, bone, cartilage, vascular structures, skin, cornea, and other soft and hard tissues [45,46,47,48,49,50]. 

One of the limitations identified in scaffolds solely made of collagen is their poor mechanical properties; therefore, crosslinking techniques have been used to improve mechanical strength. However, excessive crosslinking has been found to impair collagen’s biological activities, which renders it unsuitable for the regeneration of soft tissues [51,52]. Furthermore, collagen bioinks have low printability. Incorporating synthetic/semi-synthetic materials along with collagen to form hybrid scaffolds has been shown to improve the printability of collagen bioinks and enhance the mechanical properties of the scaffolds [16,53].

Gelatin, a product of collagen hydrolysis, is well known for its biodegradability, low immunogenicity, and being a cost-effective polymer. It has been identified that gelatin maintains the arginine-glycine-aspartic acid (RGD) sequences present in collagen, which helps cellular proliferation, adhesion, and differentiation [54]. Semi-synthetic gelatin methacrylate (GelMA) hydrogel was formulated by Van Den Bulcke et al. (2000); adding methacrylic anhydride (MA) to gelatin replaces the free amine groups in gelatin with methacrylic groups producing GelMA. Due to the existence of these methacrylic groups, gelatin can be crosslinked in the presence of a photoinitiator and light to form stable hydrogels [55]. Moreover, the produced GelMA is found to maintain the RGD motifs of gelatin; therefore, it is suitable for bioengineering applications [56]. GelMA has been identified as a desirable biomaterial for various hard and soft tissue engineering applications [57].

Properties of GelMA have been found to be enhanced by combining it with natural biopolymers such as silk fibroin, collagen, hyaluronic acid, and dextran [58]. 3D-printed hybrid hydrogels comprising GelMA with acid-soluble bovine collagen were found to have good crosslinking, improved mechanical properties, and cellular proliferation compared to GelMA hydrogels [59,60]. Properties of ovine collagen-GelMA hybrid scaffolds in tissue engineering applications have not been investigated yet.

In this study, either bovine or ovine collagen was mixed with GelMA to form hybrid hydrogel inks, which were printed using an extrusion-based printer. The printing parameters were optimized to identify the maximum percentage of bovine/ovine collagen that can be integrated with GelMA. The printed meshes were also characterized, and their properties were compared. The present study would open avenues for research to determine the suitability of these hybrid hydrogels to be used for various tissue engineering applications.

## 2. Materials and Methods

Type A porcine skin gelatin (strength ~300 g Bloom), methacrylic anhydride (MA), deuterium oxide (D_2_O) of isotopic purity 99.9 atom % D containing 0.05 wt. % 3-(trimethylsilyl)propionic-2,2,3,3-d4 acid, sodium salt, and phosphate buffered saline (PBS) tablets pH 7.4 were purchased from Sigma-Aldrich, New Zealand. Collagen from bovine hide and ovine skin were provided by New Zealand Leather and Shoe Research Association (LASRA). Allevi 2 3D bioprinter with dual extruders, needles (25 G and 27 G) used for printing, and lithium phenyl-2,4,6-trimethyl-benzoyl phosphinate (LAP) were purchased from Allevi, Philadelphia, USA. All other chemicals were of analytical grade.

### 2.1. Preparation and Characterization of GelMA

#### 2.1.1. Preparation of GelMA

**Figure 1:**
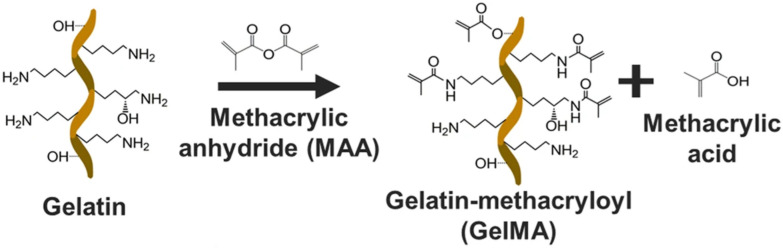
The reaction of gelatin with methacrylic anhydride [62] CC BY 4.0.

Gelatin methacrylate (GelMA) was prepared using a method reported by Van Den Bulcke et al. (2000) [54]. Synthesis of GelMA followed a direct reaction of gelatin with Methacrylic anhydride (MA), wherein gelatin was first dissolved in PBS (1×, pH 7.4) at 50 °C, after which 0.6 g MA per gram of gelatin was added dropwise at the rate of 0.5 mL/min. The mixture was left at 50 °C for 3 h with continuous stirring to allow for the substitution reaction of the methacryloyl groups onto the amino acid residue’s amine and hydroxyl groups [54,61]. Figure 1 shows the reaction synthesizing GelMA.

**Figure 2:**
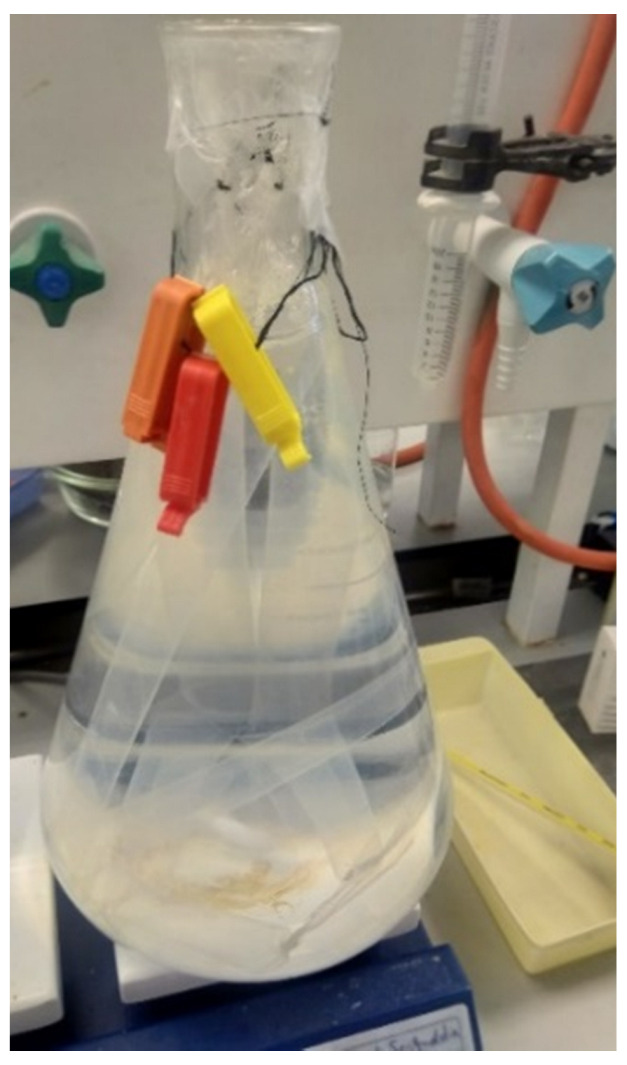
Dialysis of GelMA against DI at 50 °C for 1 week; three dialysis bags are shown in this figure.

The mixture was then centrifuged at 100 *g* for 2 min to remove excess methacrylic acid, obtained supernatant was diluted 1:1 with PBS, and the solution was dialyzed against deionized water (DI) at 50 °C using a cellulose dialysis tubing for 1 week, as shown in Figure 2. During this period, DI was changed twice every day to remove impurities such as unreacted MA and byproducts. Finally, the pH of the solution was adjusted to 7.4, after which it was freeze-dried. The GelMA obtained was stored at −80 °C until further use.

#### 2.1.2. Characterization of GelMA by NMR Spectroscopy

Using the ^1^H-NMR spectroscopy, the amount of methacrylate and methacrylamide groups present in the synthesized GelMA was determined. The NMR spectra were obtained using a spectrometer, which was fitted with a 5 mm probe type: PA BBO 400S1 BBF-H-D-05 Z, operating at the speed of 400.13 MHz and equipped with the TopSpin 3.5b.91.pl.7 software. NMR samples were prepared by dissolving 20 mg of the synthesized GelMA or gelatin from porcine skin in 1 mL of deuterium oxide (D_2_O) containing 0.05% (*w/v*) of its internal reference, trimethylsilyl propanoic acid (TMSP). Measurements of the samples were procured at 40 °C. Equation (1) was used to calculate the degree of methacrylation (DM) [63].

Equation (1)

(1) $$Degree\;of\;methacrylation\;\left(DM\right)=100\times \left(1-\frac{Lysine\;methylene\;proton\;of\;GelMA\;}{Lysine\;methylene\;proton\;of\;Gelatin}\right)$$

### 2.2. Preparation of the Inks

All inks prepared were comprised of 8% GelMA and 0.5% LAP (photoinitiator), according to previous preliminary studies done. Table 1 shows the concentration of bovine or ovine collagen incorporated within the GelMA hydrogel inks in the prepared formulations.

To prepare a 4% stock solution of bovine and ovine collagen, 200 mg of bovine or ovine collagen were dissolved in 5 mL of 0.5 N acetic acid or distilled water, respectively. Inks were prepared by dissolving the lithium acylphosphinate salt (LAP) in PBS at 50 °C under stirring. After, GelMA was added, mixed well, and left on the stirrer for 20 min to completely dissolve. Bovine or ovine collagen was then added to the GelMA mixture according to the concentrations mentioned in Table 1 (pipetted from the prepared stock solution). The prepared hydrogel inks were transferred into a 10 mL syringe. The syringe was covered with aluminium foil and set by immersion in ice for 8 min and then stored overnight at 4 °C.

### 2.3. 3D printing of Hydrogel Meshes

The design of the 3D-printed meshes was previously established using SolidWorks Software, which was uploaded onto Slice3r software. This slicing software was used to convert 3D computer-aided design (CAD) models (STL file format) into an instructional format required for layer-by-layer printing (G-code). Repetier-Host software was utilized for adjusting the printing parameters, such as the layer height, extrusion pressure, and speed of printing in order to obtain continuous and smooth filaments of the different inks. Once set, Slice3r software generates a sliced G-code file, which was used by the Allevi 3D bioprinter for fabricating the meshes of all formulations. All the meshes were printed on individual glass slides covered with waterproof tape to help easy detachment of the meshes. The meshes of size 1 cm x 1 cm were printed using two different needle sizes, 25 G and 27 G. The printed meshes were cured by exposing them to light at the wavelength of 405 nm under SUNUV LED lamp for 3 min. The intensity of the light was set at 24 W. The cured meshes were placed in a vacuum oven at room temperature for 24 h to dry.

### 2.4. Characterization of the Hydrogel Meshes

#### 2.4.1. Fourier Transform Infrared (FTIR) Spectroscopy

FTIR spectra of the individual components and meshes were carried out on a Bruker Vertex 70 FTIR spectrometer (Bruker Optics, New Zealand) in order to analyze the interactions between the components. The samples were placed directly onto the ATR crystal, and spectra were collected in transmittance mode. Each spectrum was the result of the average of 32 scans performed at 4 cm^−1^ resolution and the measurements were recorded between 4000 and 750 cm^−1^ at 21 °C.

#### 2.4.2. Crosslinking Test

Printed meshes were dried in a vacuum oven for 48 h, and initial dry weight (W0) was measured using Sartorius semi-micro 5-digit balance (Mettler Toledo, New Zealand). Three meshes from each formulation were individually immersed in PBS inside a plastic tissue cassette and left for 24 h at 37 °C. After this, the meshes were removed from the PBS solution and vacuum dried for 24 h. The final dry weight (W1) of the meshes was individually measured. The degree of crosslinking of the hydrogel meshes was determined by calculating the sol fraction given in Equation (2) [64]. The sol fraction represents the percentage of the hydrogel meshes that failed to crosslink and, as a result, dissolved in the PBS.

Equation (2)

(2) $$Sol\;fraction\;\left(\%\right)=\frac{W0-W1}{W0}\times 100$$

#### 2.4.3. Optical Microscopic Examination

Three printed meshes of each formulation were visualized under an optical microscope before and after vacuum drying. A digital microscope (Leica ICC50HD-DM750, New Zealand) was used to capture the images. For each of the formulations, the pore sizes and line diameters of meshes before and after drying were measured from five different places in each mesh, using the Leica software connected to the digital microscope.

#### 2.4.4. SEM

Three dried meshes from each formulation were visualized using scanning electron microscopy (SEM). Before imaging, the meshes were coated with platinum metal using the Hitachi E-1045 Sputter Coater for 20 s. 

#### 2.4.5. Swelling Ratio Test

Three meshes from each formulation were removed from the glass slide and placed inside a plastic tissue cassette. Six cassettes were placed inside a square Petri dish, and 20 mL of PBS was added, as this volume was identified to be sufficient to completely immerse the meshes in PBS and were left for 24 h at 37 °C. Sartorius semi-micro 5-digit balance was used to measure the weights of the swollen meshes after gently blotting the meshes using tissue paper. An increase in the fractional weight of the meshes due to the absorption of PBS, also known as the swelling ratio, was calculated using Equation (3) [54,64].

Equation (3)

(3) $$Equilibrium\;Swelling\;Ratio\;\left(ESR\right)\%=\frac{\left(Sollwen\;Weight-Dry\;Weight\right)}{Dry\;Weight}\times 100$$

#### 2.4.6. Degradation Test

The initial dried weight (W0) of the meshes was measured after drying the printed meshes in a vacuum oven for 48 h. Twelve meshes from each formulation were placed inside a holder that was immersed in PBS and incubated at room temperature. At the time intervals 1, 2, 3, and 4 days, three meshes from each formulation were removed, and vacuum dried for 24 h, after which they were weighed again (W1). The degradation profile of meshes from each formulation was determined by means of calculating the gel fraction using Equation (4) [54,64].

Equation (4)

(4) $$Gel\;fraction\;\left(\%\right)=\frac{W1}{W0}\times 100$$

The gel fraction represents the percentage weight of the hydrogel meshes that remained.

#### 2.4.7. Statistical Analysis

The results of the tests were analyzed statistically using R programming and GraphPad Prism software (version 8). The data obtained from the characterization tests were subjected to Shapiro Wilcox’s test to check for normality. The statistical significance was calculated using one-way ANOVA (parametric analysis, used when data follows normal distribution)/ Kruskal–Wallis analysis (non-parametric alternative) followed by the Tukey post hoc test. The level of significance was set at *p* < 0.05.

## 3. Results and Discussion

### 3.1. Characterization of GelMA by NMR Spectroscopy

**Figure 3:**
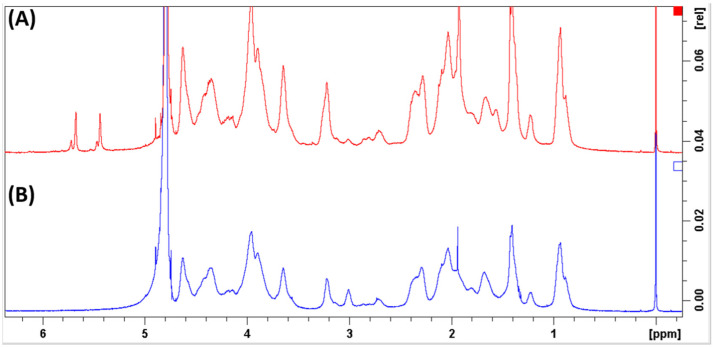
1H-NMR spectra of (**A**) GelMA, compared with (**B**) porcine gelatine in 1 mL of D2O containing 0.05% (*w/v*) TMSP.

NMR analysis confirmed the substitution of methacryolyl groups in the crosslinked GelMA meshes, as shown by the methyl proton peak of GelMA appearing at 1.9 ppm and the acrylic proton peak emerging between 5.5 and 5.9 ppm in the spectra, represented in Figure 3A. On the other hand, those peaks were absent in the gelatin spectra as represented in Figure 3B. The peak at 2.7 ppm was used for normalization, as it was found to be closer to the lysine peaks and thus was less affected by baseline and phasing errors. The degree of methacrylation of GelMA, calculated using Equation (1), was found to be 62.7%.

A decrease in free lysine (NH_2_CH_2_CH_2_CH_2_CH_2_ -) signal occurred in the gelatin sample, showed at the 3-ppm peak. The peaks at 5.9–5.5 ppm and the smaller peaks at 6.1 and 5.7 ppm have been identified to indicate the presence of the acrylic protons (CH_2_=C(CH_3_)COO-) in the methacrylamide groups and methacrylate groups, respectively. Furthermore, the peak at 1.9 ppm indicates the methyl protons (CH_2_=C(CH_3_)CO-) of the methacryloyl groups [63,65].

### 3.2. 3D printing of Hydrogel Meshes

The chosen printing parameters, represented in Table 2, such as speed and pressure, were optimized to ensure that the inks were extruded as a continuous filament.

#### 3.2.1. Effect of Gauge Size on the Extrudability of Different inks

In this study, two plastic tapered needles of different gauge sizes, namely 25 G and 27 G, were used for printing. The larger the gauge size, the smaller the inner diameter; therefore, the 27 G needles having smaller inner diameters compared to the 25 G were found to be suitable to print low viscous materials. 

The rheological properties of biomaterials, such as their viscosity, affect their printability. Especially in the case of extrusion-based printing techniques, viscosity has been shown to affect the shape of the printed meshes due to a phenomenon called spreading and drooping, which controls the extrusion of inks from the needles as either continuous filaments or as droplets [66]. Wüst et al. identified that highly viscous inks obtained upon the incorporation of hydroxyapatite into gelatin-alginate composite hydrogels were not extruded as continuous filaments but as droplets from the printing needles [67].

GelMA meshes with 1 and 2% bovine and ovine collagen were difficult to extrude from the 27 G needles since increasing the concentration of the incorporated collagen within the meshes might have resulted in an increase in viscosity due to the increase in the molecular weight of the hydrogels. Polymers with a higher molecular weight and viscosity were found to be difficult to extrude from printing needles [19,20]. G, GB0.5, and GS0.5 hydrogel inks were smoothly extruded from the 27 G needles. Printing with the 27 G needles helped obtain thin lines with well-defined pores since the inks were extruded as continuous filaments from smaller-sized needles. All the meshes were successfully printed using the 25 G needles, including the meshes containing higher concentrations of collagen. To maintain uniformity and make appropriate comparisons, meshes printed using 25 G needles for all the formulations were used in the characterization tests.

#### 3.2.2. Effect of Printing Parameters on the Extrudability of the Inks

The meshes of all the formulations were printed at a temperature of 20 °C (± 2 °C). The pressure required for printing the meshes, to a great extent, depends on the temperature during printing, rheological properties of the ink, and the needle size. These factors also affect the extrudability and consistency of the filaments [68].

GelMA meshes with 0.5% bovine collagen (GB0.5) required lower pressure (6 PSI) to print compared with GS0.5 (18 PSI), printed at the same speed of 6 mm/s using 25 G needles. Similarly, GelMA with 0.5% ovine collagen (GS1) required lower pressure of 11 PSI and was printed at the speed of 6 mm/s when compared with higher pressure (16.2 PSI) required by GB1 inks printed at the speed of 4 mm/s. The lower pressure required to extrude the inks with 0.5% and 1% bovine collagen printed at a higher speed might be due to the presence of acetic acid used to dissolve the bovine collagen, which might have lowered its viscosity when compared with the deionized water used to dissolve the ovine collagen. The addition of acetic acid to collagen was found to decrease the viscosity of the solution [69].

Increasing the concentration of bovine collagen to 1% increased its viscosity; thus, when compared with GB0.5 meshes, higher pressure was required to print GB1 meshes at the same speed of 6 mm/s. This higher printing pressure can be attributed to the increased viscosity of the GB1 ink, associated with its higher molecular weight. This was in compliance with the study by Naghieh et al., which showed that hydrogels with high viscosity require higher pressure for printing and vice versa [20].

GB0.5 meshes printed using 27 G needles required lower pressure and speed compared to GS0.5 meshes using the same needle size. Printing with smaller diameter (27 G) needles resulted in smooth, consistent, and thin lines for inks incorporated with lower concentrations of collagen. Furthermore, both GB0.5 and GS0.5 inks printed with 27 G needles required a higher pressure for printing than with 25 G needles; this is because printing with a smaller needle diameter size requires higher pressure to allow for the extrusion of the ink from the small orifice. Similarly, G inks printed with 25 G needles required a lower pressure and were printed faster compared with 25 G needles. The primary drawback of GelMA meshes was identified to be its lower viscosity at room temperature, which affects the printability of GelMA hydrogels [70]. The addition of natural polymers, such as hyaluronic acid, to form hybrid hydrogels has been found to increase the viscosity of inks compared to hydrogels solely comprised of GelMA [66].

Inks with higher concentrations, 2% of bovine and ovine collagen (25 G needle), although requiring lower pressure for printing, were found to have a tendency to spread. This could have been due to the high viscosity of inks with higher molecular weight, which was found to increase with the concentration of collagen. The higher concentrations of collagen incorporated in these inks resulted in the spreading of the printed hybrid inks lines; thus, these meshes were found to have poor shape fidelity. Shape fidelity, described as the ability of the hydrogel meshes to maintain their shape, is an important characteristic expressing the printability of hydrogel inks. Hydrogel inks are said to have good or poor fidelity depending on their ability to maintain the diameter of the extruded filament after printing, stability of the pore shape and size of the printed meshes and the inability of the printed structure to collapse [71].

### 3.3. Characterization of the Hydrogel Meshes

#### 3.3.1. Fourier Transform Infrared (FTIR) Spectroscopy

**Figure 4:**
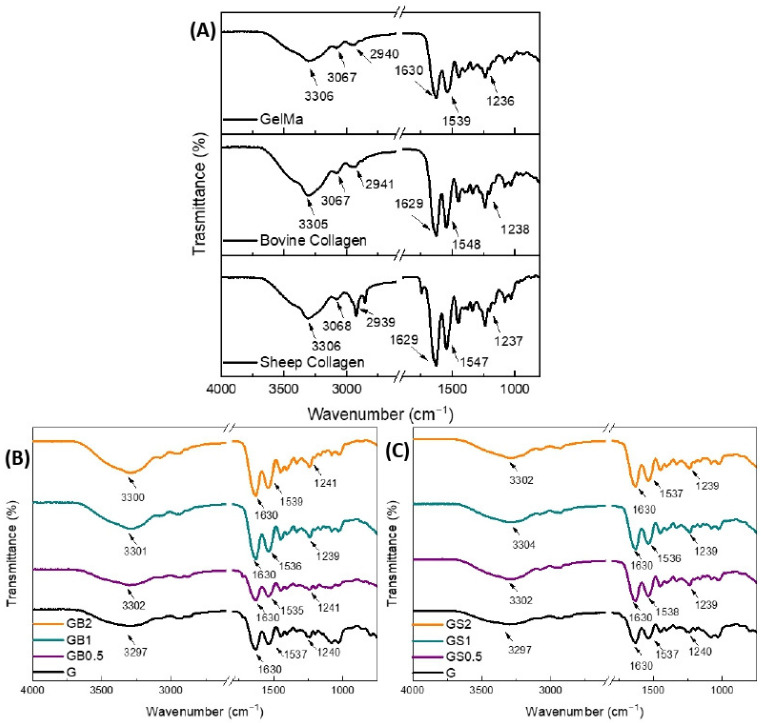
FTIR spectra of (**A**) the individual components, (**B**) the GelMA meshes containing 0.5%, 1%, and 2% bovine collagen compared with GelMA meshes, and (**C**) the GelMA meshes containing 0.5%, 1%, and 2% ovine (sheep) collagen compared with GelMA meshes.

FTIR spectra of the individual components and printed meshes are shown in Figure 4. The FTIR spectra of GelMA and collagen (bovine and ovine) showed the typical bands of proteins: the broadband positioned at 3306 cm^−1^ was associated with O-H and N–H stretching; the bands at 2924 and 2850 cm^−1^ represented the C-H stretching; and the bands at 1630, 1537/1547, and 1236 cm^−1^ were related to the C=O stretching (amide I), N–H bending (amide II), and C–N stretching and N–H bending (amide III), respectively [72].

It is known that modified gelatin obtains the feature of photo-crosslinking due to the presence of methacryloyl groups. Therefore, it was expected that GelMA would crosslink under UV light when in the presence of the photoinitiator LAP [73]. The photopolymerization of GelMA (Figure 4(B)/(C)-G) resulted in some shifts in the main bands of the protein spectra compared to the individual component (GelMA), which were related to the photo-crosslinking of GelMA.

The absence of methacryloyl groups in the collagen incorporated in GB0.5, GB1, GB2, GS0.5, GS1, and GS2 formulations forewarned the non-participation of collagen (bovine and ovine) in the photopolymerization reaction. Moreover, the addition of collagen to GelMA might have hindered the photopolymerization reaction and/or could have led to some physical interactions between the proteins. However, lower physical interactions were observed with ovine collagen when compared with bovine collagen indicating a better-crosslinked structure of ovine hybrid hydrogels. These interactions might explain the shifting of the O-H, amide II, and amide III bands after the incorporation of collagen into the formulation Those interactions and hindering of the photopolymerization reaction might have an effect on the crosslinking and degradation properties of the printed meshes [74].

#### 3.3.2. Crosslinking Test

Photo-polymerization, a process responsible for the formation of crosslinks within the polymer network, allows for the development of stable hydrogel meshes by controlling the gelation process. Polymerization of GelMA under the presence of UV light and an aqueous soluble photo-initiator such as LAP can help stabilize the structure of the printed meshes [61,75,76]. Studies have reported that LAP used for crosslinking GelMA scaffolds promotes high cellular viability compared to other photo-initiators [77].

Increased strength of hydrogels is said to be associated with a higher degree of crosslinking. However, this is only until the hydrogel reaches its optimum crosslinking degree [52,78]. Increased strength and a higher degree of crosslinking were found to be suitable for greater cellular interactions and proliferation within the hydrogels [79,80]. Crosslinking of the hydrogel meshes depends upon several factors, such as the amount of photo-initiator added, the type used, and curing intensity and time [61].

The sol fraction (%) was calculated using Equation (2). It represents the percentage of polymer that did not crosslink during the photopolymerization reaction [81]. Thus, the lower the sol fraction, the more the hydrogel meshes had crosslinked.

**Figure 5:**
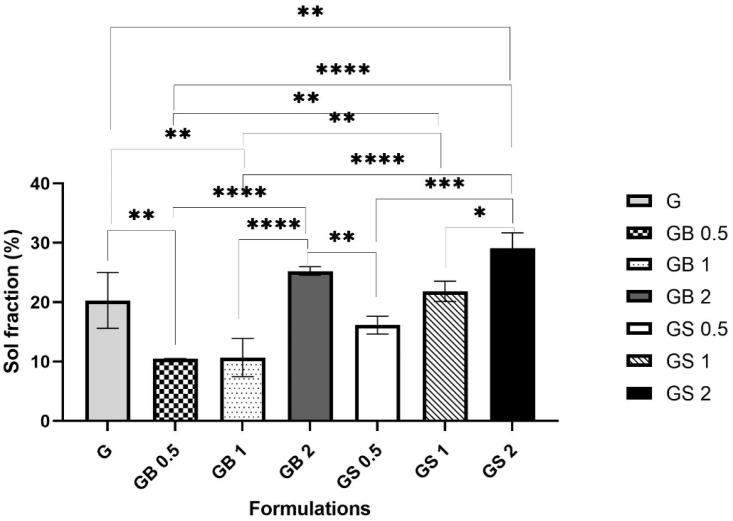
Comparison of sol fraction (%) between the meshes of different formulations. (**** *p* < 0.0001, *** *p* < 0.001, ** *p* < 0.01, * *p* < 0.05).

In this study, the sol fraction (%) of the different hydrogel formulations G, GB0.5, GB1, GB2, GS0.5, GS1, and GS2 were compared as shown in Figure 5. Incorporating 0.5% and 1% bovine and 0.5% ovine collagen resulted in decreased sol fractions (%) of 10.47 ± 0.04%, 10.67 ± 3.2%, and 16.14 ± 1.4%, respectively, when compared with the G meshes (20.31 ± 4.7%), indicating a higher degree of crosslinking in GB0.5, GS0.5, and GB1 meshes. 0.6% collagen in GelMA hybrid scaffolds and 1% concentration of collagen in collagen- glycose aminoglycan scaffolds were found to be associated with a higher degree of crosslinking, and increased cell metabolic activity [59,82]. Integrating polymers within GelMA meshes was found to help increase the degree of crosslinking and form hybrid scaffolds, which are suitable for bone tissue regeneration [76].

However, with a higher concentration of 2% bovine collagen, 1%, and 2% ovine collagen, the hydrogel meshes were found to have an increased sol fraction (%) of 25.26 ± 0.7%, 21.82 ± 1.7%, and 29.07 ± 2.6% respectively, indicating a lower degree of crosslinking when compared with G meshes. The addition of collagen to form hybrid hydrogels hindered the photo-crosslinking of the GelMA, as seen confirmed by FTIR analysis, which might have decreased the degree of crosslinking in the meshes incorporated with higher concentrations of collagen.

Bovine meshes of all three concentrations were found to be associated with a higher degree of crosslinking indicated by their lower sol fraction (%) when compared with ovine meshes. FTIR analysis showed better crosslinking of ovine hybrid meshes compared to bovine hybrid meshes, which would indicate enhancement of the crosslinking of the printed structure. However, the water-soluble nature of ovine collagen could have caused the meshes to be more susceptible to degradation in PBS, resulting in a higher sol fraction (%). This is supported by the associations of higher sol fraction (%) with the water-soluble nature of collagens and decreased sol fraction (%) with acid-soluble collagens identified [35,37,83].

#### 3.3.3. Optical Microscopic Examination

**Figure 6:**
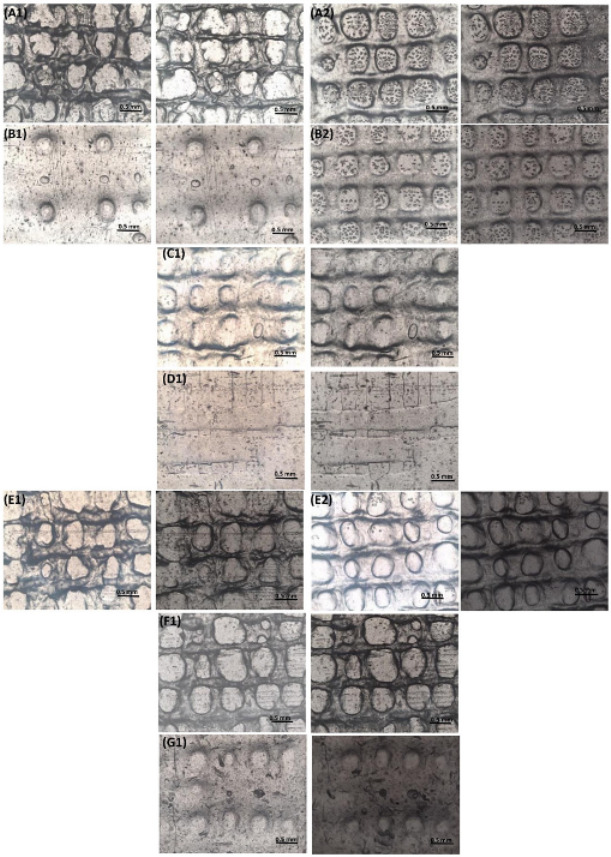
Microscopic images of the meshes after printing (left for each group) and after vacuum drying (right of each group) of the formulations, scale bar = 0.5 mm (**A)** G, (**B**) GB0.5, (**C**) GB1, (**D**) GB2, (**E**) GS0.5, (**F**) GS1, and (**G**) GS2, where 1 refers to meshes printed with 25 G and 2 refers to those printed with 27 G needles.

GelMA hydrogels and GelMA hybrid hydrogels with different concentrations (0.5%, 1%, and 2%) of bovine and ovine collagen were visualized under the microscope using 4× magnification. Figure 6 highlights the meshes printed with both 25 G and 27 G needles, which were visualized after printing (before drying), and after vacuum drying, for each formulation.

**Figure 7:**
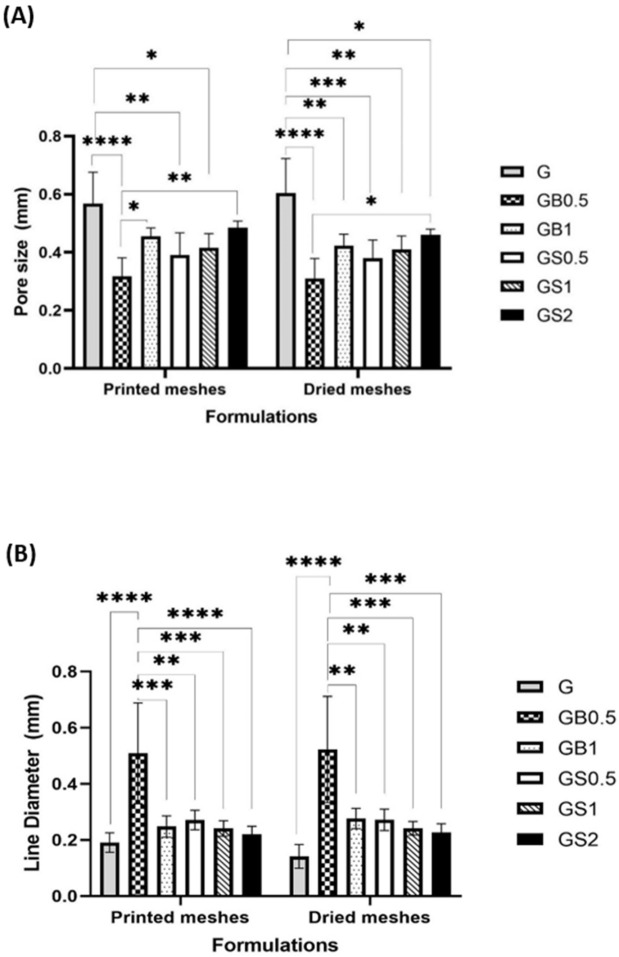
Comparison of (**A**) pore size and (**B**) line diameter of all the meshes before and after vacuum drying, printed using 25 G needles (**** *p* < 0.0001, *** *p* < 0.001, ** *p* < 0.01, * *p* < 0.05).

Images obtained of the meshes printed with 27 G needles showed smoother printed lines and well-defined pores when compared to the meshes printed using 25 G needles. GelMA meshes printed with 25 G needles had poorly printed lines due to the increased thickness of the extruded filament. The high viscosity of GS2 inks was difficult to extrude resulting in an inconsistent filament, due to which the lines of these meshes were not printed properly. Figure 7A,B highlights the pore size and line diameter of printed and dried meshes of all the formulations. There was no significant difference in the pore size and line diameter when comparing each formulation before and after drying. However, there were statistically significant differences between both the pore size and line diameter of different formulations when comparing each formulation with other formulations.

The line diameter was found to be inversely associated with the pore size of the hydrogel meshes; this is because the space occupied by thicker printed lines reduces the pore size of the printed mesh. Both printed and dried GB0.5 meshes were found to have larger diameter lines and smaller pores than other formulations. Thicker lines of GB0.5 resulted due to lower viscosity of the ink. Acetic acid added to dissolve the bovine collagen was found to reduce the viscosity of the inks [69]. However, viscosity is found to increase by integrating GelMA hydrogels with natural polymers [66]. An increase in the degree of crosslinking has been found to be associated with smaller pore sizes due to the formation of highly dense meshes [84]. Although the degree of crosslinking was identified to be similar for GB0.5 and GB1 meshes, the incorporation of 1% bovine collagen increased the pore size of the meshes. This is due to the higher molecular weight associated with increased concentrations of collagen, thus increasing the viscosity of the inks. This allowed for higher printing fidelity and thinner printed lines, which increased the pores size of GB1 meshes. Research by Cooney et al. identified that concentration of the polymer does not influence the pore size of the hydrogels; however, the fabrication technique including the designed printed structure has a strong influence on the pore size [85].

GS0.5 meshes were found to have larger pore sizes and thinner line diameter when compared to the GB0.5 meshes, which might be due to its higher viscosity associated with the water-soluble nature of ovine collagen (as it is not dissolved in acetic acid). GS1 and GB1 meshes were found to have similar line sizes and larger pore diameters. The pore size of these meshes was found to be smaller than GelMA meshes, and larger than GB0.5 meshes. GS2 and GB2 meshes were found to have either smaller pore size and larger lines and complete absence of pores, respectively, when compared with the meshes with 1% concentration of collagen. This could be due to the poor shape fidelity of meshes with higher concentrations of collagen, as discussed above.

When compared with GelMA-only meshes (G), collagen-incorporated meshes were found to have lower pore size and higher line diameter. It was found that the suitability of the meshes in different applications depends on the size of the pores and the cells involved. Smaller pore size is required by smaller cells for adhesion and migration, and vice versa [86,87].

#### 3.3.4. SEM

**Figure 8:**
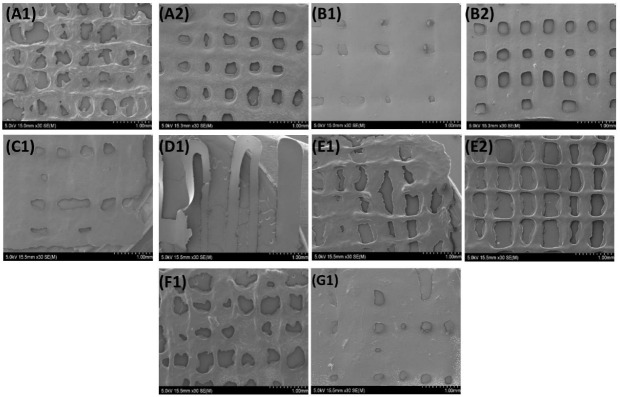
SEM images of the meshes printed with two needles of the formulations, scale bar = 1 mm (**A**) G, (**B**) GB0.5, (**C**) GB1, (**D**) GB2, (**E**) GS0.5, (**F**) GS1, and (**G**) GS2, where 1 refers to meshes printed with 25 G, and 2 refers to those printed with 27 G needles.

Phase-separation of polymers in hybrid hydrogels is found to have an influence on the rheological properties of the hydrogels; the absence of which indicates uniformity in the properties within each of the meshes [88]. SEM images of the meshes revealed the absence of any phase-separation between the components of all the formulations, and homogeneity of the inks, as highlighted in Figure 8. This goes in hand with the unique advantage of extrusion-based bioprinting, which ensures the homogenous distribution of the material being printed [13,14,15].

Meshes printed with 27 G needles had well-defined, consistent pores and lines when compared with meshes printed with 25 G needles. However, this was only for inks of lower viscosity, due to difficulties printing highly viscous inks with smaller diameter (27 G) needles.

Incorporating collagen helped obtain hybrid hydrogel meshes with a smoother surface when compared with GelMA meshes. The surface topology of meshes is required to support the application of the meshes in various tissues of the human body. The smooth surface of meshes has been identified to be associated with a low frictional coefficient. The movement of different joins, eyelids, and other such functions require low friction between the surfaces [89]. Such scaffolds have been developed for the regeneration of temporomandibular joints, cartilage, and meniscus tissues [90,91,92]. Thus, the smooth surface of collagen/GelMA hybrid hydrogels meshes obtained might be beneficial in these applications.

#### 3.3.5. Swelling Ratio Test

**Figure 9:**
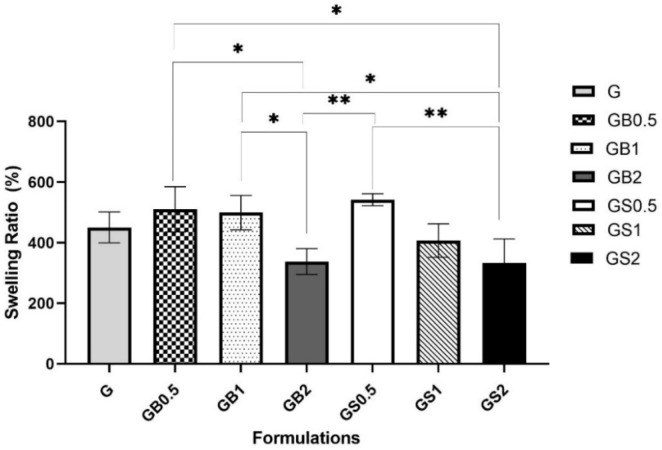
Comparison of the swelling ratio (%) calculated for meshes of different formulations (** *p* < 0.01, * *p* < 0.05).

Fractional weight gain of the hydrogels due to water absorption defines the swelling ratio [81]. The swelling ratio is an important characteristic of hydrogels, as it has been identified to help determine the diffusion of waste and nutrients in and out of any cells cultured on the matrix [65]. The swelling ratio is dependent on the amount of photo-initiator added, the degree of crosslinking, and also the architecture of the printed meshes. A higher degree of crosslinking has been found to be associated with lower swelling ratios, which has also been identified to increase the stiffness of the hydrogels [61,76,93]. The swelling ratio (SR) (%) of the meshes was calculated using Equation (3) and is represented in Figure 9. The results were found to be statistically significant.

The incorporation of 0.5% and 1% of bovine collagen resulted in increased swelling ratios, 510.03 ± 74.6% and 499.21 ± 56.7%, respectively, when compared with GB2 meshes (337.89 ± 42.6%) and G meshes (450.50 ± 50.8%). Higher swelling ratios are generally associated with weaker physical interactions [94]. Decreased swelling ratios of meshes with higher concentrations could have been due to the appearance of hydrogen bonds as identified by the shifts in FTIR spectra, which reduce the water uptake capacity of these meshes. Water absorption kinetics of hydrogels are found to depend on the crosslinking reactions and the physical interactions involved with the development of hydrogel structure [95].

Similar results were observed with 0.5% ovine collagen in hybrid meshes (541.66 ± 19.9%) when compared with GelMA. GS1 meshes were found to have a lower swelling ratio of 407.17 ± 55.4% than G and GB1 meshes. Higher crosslinked structure of GS1 meshes when compared with GB1 meshes, as identified from FTIR analysis; thus, showing increased swelling ratios of GS1 collagen. Tierney et al. (2009) showed that incorporating 1% collagen in hybrid hydrogels helped to improve the crosslinking degree of the hybrid hydrogels [82]. However, higher swelling ratios were obtained with GB1, as the meshes incorporated with bovine collagen were found to have higher physical interactions hindering their structural crosslinking. Similarly, GS2 meshes were found to have a lower swelling ratio (332.76 ± 79.3) than GB2 meshes. The SR (%) decreased upon increasing the concentration of both bovine and ovine collagen within the GelMA meshes. This could be further correlated with the loss of gel fraction (%), which was higher in GB2 and GS2 meshes, thus meshes degraded faster, as identified from the degradation tests of the meshes, discussed later.

The incorporation of collagen was found to improve the mechanical strength of the hybrid hydrogels and was suitable in cartilage, bone, and corneal tissue engineering applications [96,97,98,99]. Further studies testing the mechanical properties of meshes are required to understand their potential in different applications.

#### 3.3.6. Degradation Test

Degradable biomaterials, producing non-cytotoxic and biocompatible byproducts are preferred for the regeneration of tissues, as this ensures no interference of the materials with the growth of the new tissue. The desired degradation rates are said to depend on the time taken for regeneration of the damaged tissue [100]. Degradation is found to enhance the behavior of cells, such as better proliferation within its extracellular matrix, that is, the tissue-engineered construct. The degree of crosslinking has been found to have an influence on the degradation rates of hydrogel polymers [101]. The gel fraction (%), calculated using Equation (4) gives the amount of polymer remaining after degradation. The higher the crosslinking degree, the lower the gel fraction (%).

**Figure 10:**
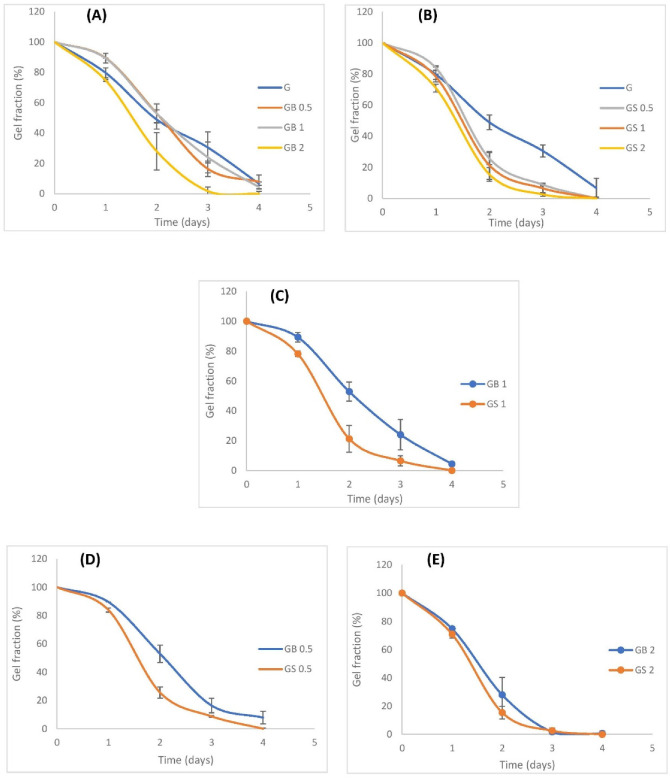
Degradation rates of hydrogel meshes in PBS (pH 7.4), (**A**) comparison of G with different concentrations (0.5%, 1%, 2%) of bovine collagen/GelMA hybrid meshes, (**B**) comparison of G with different concentrations (0.5%, 1%, 2%) of ovine collagen/GelMA hybrid meshes, (**C**–**E**) compare 0.5%, 1%, and 2% bovine and ovine collagen, respectively (**** *p* < 0.0001).

In this study, GB0.5 and GB1 hydrogel meshes incorporated with bovine collagen, initially had higher gel fraction values compared to G hydrogel meshes on days 1 and 2, after which the levels dropped on day 3. Increased concentrations of collagen in hydrogels were found to have smaller fiber lengths, decreased fiber organization, and slower degradation rates [102,103]. Faster degradation of G hydrogels on days 1 and 2 might be due to the lower mechanical strength of these hydrogels [104]. Furthermore, the lower degradation of the hybrid GB0.5 and GB1 hydrogel meshes goes in line with the results of another study where hybrid meshes incorporated with 0.6% and 1% of bovine hide collagen were found to have low degradation rates compared to non-hybrid hydrogel meshes [59,82]. However, GB2 meshes degraded at a faster rate compared to G, GB0.5, and GB1 meshes. The faster degradation of GB2 hybrid meshes was probably due to the high concentration of collagen within the hydrogel matrix that might have hindered the ability of GelMA to crosslink. Figure 10A compares the GelMA meshes with hybrid hydrogel meshes incorporating different concentrations of bovine collagen. 

Figure 10B compares the degradation rates of hybrid hydrogel meshes incorporated with 0.5, 1, and 2% ovine collagen with GelMA meshes. Initially, on day 1, GS0.5 meshes had a higher gel fraction than GelMA meshes. However, from day 2, the meshes degraded at a faster rate as shown by their lower gel fraction values (%). All the meshes with ovine collagen had lower rates of degradation than GelMA meshes. Upon comparing the meshes containing the same concentrations of ovine and bovine collagen as shown in Figure 10C–E, ovine collagen meshes degraded much faster, except in the case of 2% bovine and ovine collagen meshes, which had similar degradation rates. This higher degradation rate of ovine collagen meshes could have been due to its higher water solubility when compared to bovine collagen. Research showed that rapid degradation rates of water-soluble polymers are found to be suitable for promoting the proliferation of fibroblasts, and beneficial in bone tissue engineering applications [38,105]. However, very high degradation rates of scaffolds are not preferred as such scaffolds fail to support the regenerating tissue. On the other hand, scaffolds with very low degradation rates could interfere with the new tissue formation [106]. Hence, further proliferation studies of specific cells on these meshes are advised to determine the suitability of bovine and ovine hybrid hydrogel scaffolds in specific applications.

Almost all the meshes were degraded completely by day 4. Only G, GB0.5, and GB1 meshes had a gel fraction (%) of 6.6 ± 6.2, 7.8 ± 4.4, and 4.3 ± 1.1, respectively, on day 4. Stable degradation rates were seen in GB1 meshes, which were found to be essential to determining the functioning of the meshes in tissue engineering applications [101].

## 4. Conclusions

In this research, GelMA hydrogel meshes and hybrid hydrogel meshes comprising of GelMA with 0.5%, 1%, and 2% bovine/ovine collagen were printed upon optimizing the printing parameters, and the characteristics of the meshes were determined by studying their swelling ratio, degree of crosslinking, and degradation rates, by examining the physical and chemical properties using optical microscopy, SEM, and FTIR. The research successfully demonstrates the improved printability of collagen when combined with semi-synthetic GelMA. The maximum percentage of collagen that could be incorporated into the GelMA/Collagen hybrid scaffolds was determined as 1%, as these meshes showed good printability, stable degradation rates, higher crosslinking, and lower swelling ratios. Printing meshes incorporated with 2% collagen resulted in poor shape fidelity and a lower degree of crosslinking.

Upon comparing the properties of the lower concentrations (0.5% and 1%) of bovine hybrid scaffolds with ovine hybrid scaffolds, bovine hybrid scaffolds were found to have a lower sol fraction (%) and slower degradation rates. Although FTIR analysis showed better crosslinking in the structure of ovine meshes, the water-soluble nature of the ovine collagen increased the susceptibility of these hydrogels to faster degradation in PBS. They could be suitable in bone tissue engineering applications; however, this depends on the mechanical properties of the meshes which need to be tested. Similar swelling ratio and consistency of the extruded filament of lower concentrations of bovine and ovine hybrid hydrogels were observed. All the meshes with collagen incorporated had a smooth surface which is beneficial in temporomandibular joint, cartilage, and meniscal tissue engineering applications.

In conclusion, incorporating 1% bovine collagen helped obtain smooth meshes with a high degree of crosslinking and consistent degradation rates, which ensures proper functioning of meshes in various tissue engineering applications involving joint bone, cartilage, cornea, and many soft tissues. Further studies determining the effect of cellular growth and viability on these meshes are essential to identify their suitability in specific applications.

## Figures and Tables

**Table 1:** Formulation of different inks prepared for printing (“-“ referring to Nil).

No	Formulations	Bovine Collagen	Ovine Collagen
1	G	-	-
2	GB0.5	0.5%	-
3	GB1	1%	-
4	GB2	2%	-
5	GS0.5	-	0.5%
6	GS1	-	1%
7	GS2	-	2%

**Table 2:** Optimized printing parameters for each formulation.

Composition	Nozzle (G)	Speed (mm/s)	Pressure (PSI)
G	25 27	5 3	9.8 10.5
GB0.5	25 27	6 4	6 8
GB1	25	6	11
GB2	25	4	6.5
GS0.5	25 27	6 6	18 22.3
GS1	25	4	16.2
GS2	25	4	6.5

## Data Availability

The data presented in this study are available in this article.
